# Graphene-supported SnO_2_ nanoparticles prepared by a solvothermal approach for an enhanced electrochemical performance in lithium-ion batteries

**DOI:** 10.1186/1556-276X-7-215

**Published:** 2012-04-13

**Authors:** Bei Wang, Dawei Su, Jinsoo Park, Hyojun Ahn, Guoxiu Wang

**Affiliations:** 1 Centre for Clean Energy Technology, School of Chemistry and Forensic Science, University of Technology, City Campus, Broadway, Sydney, NSW, 2007, Australia; 2 School of Materials Science and Engineering, Gyeongsang National University, 900 Gazwa-dong, Jinju, Gyeongnam, 660-701, South Korea

**Keywords:** SnO_2_, Graphene nanosheets, Nanocomposite, Lithium-ion batteries

## Abstract

SnO_2_ nanoparticles were dispersed on graphene nanosheets through a solvothermal approach using ethylene glycol as the solvent. The uniform distribution of SnO_2_ nanoparticles on graphene nanosheets has been confirmed by scanning electron microscopy and transmission electron microscopy. The particle size of SnO_2_ was determined to be around 5 nm. The as-synthesized SnO_2_/graphene nanocomposite exhibited an enhanced electrochemical performance in lithium-ion batteries, compared with bare graphene nanosheets and bare SnO_2_ nanoparticles. The SnO_2_/graphene nanocomposite electrode delivered a reversible lithium storage capacity of 830 mAh g^−1^ and a stable cyclability up to 100 cycles. The excellent electrochemical properties of this graphene-supported nanocomposite could be attributed to the insertion of nanoparticles between graphene nanolayers and the optimized nanoparticles distribution on graphene nanosheets.

## Background

Graphene has been emerged as a rising star in materials science and as an excellent candidate for many applications due to its unique two dimensional (2D) nanostructure [1], outstanding electrical properties [2], and ultrahigh specific surface area [3]. The applications of graphene include gas molecule adsorption [4], quantum dots [5], transistors [6], lithium-ion batteries [7], supercapacitors [8], lithium air batteries [9], and drug delivery [10]. In particular, graphene has attracted worldwide attention for energy storage and conversion. With the formation of sandwich-like three dimensional (3D) nanostructured composite materials, the restacking of graphene nanosheets (GNS) can be effectively prevented and therefore the electrochemical properties of the nanocomposite electrodes could be significantly improved by using nanocrystallines to insert between layers of graphene nanosheets [3,11].

SnO_2_ has been examined as an anode material for lithium-ion batteries with a high theoretical capacity of 782 mAh g^−1^[12]. SnO_2_ forms metal alloys when reacting with lithium, leading to reversible transformations between lithium tin alloys (Li_x_Sn) and tin metal when the lithiation and delithiation proceed. However, the capacity of bulk SnO_2_ electrode fades quickly during prolonged cycling [13]. To further improve the electrochemical performance and the cycle life of SnO_2_ electrodes for long-term cycling, one approach is to synthesize nanosized SnO_2_ crystals with different morphologies, such as nanowires [14], nanotubes [15], and mesoporous structure [16]. These nanostructured SnO_2_ materials were reported to deliver greatly enhanced specific capacities with durable cycling stabilities. In order to mechanically buffer the volume expansion associated with the charge/discharge processes in the lithium-ion cells, the formation of SnO_2_/graphene nanocomposites has also been proved to be feasible. Many methods have been implemented to distribute SnO_2_ nanocrystals on graphene nanosheets, including *in situ* chemical preparation [13,17], reassembling process [18], gas–liquid interfacial synthesis [19], as well as hydrothermal and solvothermal methods [20,21].

In this paper, we employ a facile solvothermal technique to disperse SnO_2_ nanoparticles with a controlled size on graphene nanosheets. The as-prepared SnO_2_/GNS nanocomposite showed uniform SnO_2_ nanoparticles distribution and significantly improved electrochemical properties, compared with bare graphene nanosheets and SnO_2_ nanoparticles. The solvothermal approach developed in this investigation could be used for the synthesis of other metal oxide/graphene nanocomposites.

## Methods

Graphene oxide (GO) powders were prepared via a chemical approach derived from Hummers' method [22], according to the previously reported procedure [7]. In a typical synthesis process, 40 mg GO was firstly dispersed in 40 ml ethylene glycol by ultrasonification for 1 h, followed by the addition of 0.1 mmol SnCl_2_·2H_2_O powders. The mixture was vigorously stirred for half an hour, and then transferred to a 50 ml Teflon lined autoclave, which was sealed and maintained in an oven at 160°C for 6 h. Afterwards, the black precipitates (SnO_2_/GNS) were collected, washed with deionized water and ethanol to remove the impurities, and isolated by vacuum filtration. The product was then dried in a vacuum oven at 60°C, and further sintered at 300°C for 4 h in argon to increase the crystallinity. For the comparison, bare SnO_2_ nanoparticles were also synthesized by the same experimental procedure without the presence of GO in the mixture solution.

The X-ray diffraction (XRD) pattern of the as-synthesized material was measured using a Siemens D5000 X-ray diffractometer (Siemens Company, Wittelsbacherplatz 2, Munich, Germany) from 10° to 80° under a scan rate of 1° min^−1^. The Raman measurement of the SnO_2_/GNS nanocomposite was conducted on a confocal Micro Raman Spectrometer with LabRAM HR system (HORIBA Korea Ltd., Pucheon, Kyunggido Korea) using a 632.8 nm He-Ne laser source. The spectra were recorded in the range of 200 to 2,000 cm^−1^ with accumulated scans for an enhanced resolution. Field emission scanning electron microscope (FESEM) observations were performed using a Zeiss Supra 55VP FESEM with an Oxford energy dispersive spectrometry system (Carl Zeiss Nanotechnology Center, Oberkochen, Germany). The transmission electron microscopy (TEM) analysis was carried out using a Jeol 2011 TEM facility (JEOL Ltd., Tokyo, Japan). The graphene (carbon) content in the composite material was determined by thermogravimetric analysis (TGA) on a TGA/DTA analyzer (TA Instruments, SDT 2960 module, New Castle, DE, USA) in air at 10°C min^−1^ ranging from room temperature to 1,000°C.

CR2032 coin cells were assembled in an argon-filled glove box (Unilab, M Braun Inertgas-Systeme GmbH, Garching, Germany) in which the levels of moisture and oxygen were controlled to be less than 0.1 ppm. The electrodes were made by mixing 80 wt% SnO_2_/GNS active materials, 10 wt% carbon black, and 10 wt% polyvinylidene fluoride binder in the *N*-methyl-2-pyrrolidone solvent to form a slurry. Then, the slurry was coated on copper foil substrate. Lithium foils were used as the negative electrodes. The electrolyte was 1 M LiPF_6_ in ethylene carbonate and dimethyl carbonate (1:1). Cyclic voltammetry (CV) tests were carried out on an electrochemistry workstation (CHI660D, CH Instrument, Inc., Austin, TX, USA) at a scan rate of 0.1 mV s^−1^ vs. Li/Li^+^ reference electrode in the voltage range of 0.01 to 3 V. Galvanostatic charge/discharge measurements were conducted on the Neware battery tester (Neware Co.,Ltd., Shenzhen, China) with a current rate of 0.1 C for 100 cycles. Electrochemical impedance spectroscopy was performed on the same electrochemistry workstation. The frequency was set in 0.01 Hz–100 kHz with the amplitude of 5 mV. The charge/discharge performance was also investigated for bare graphene nanosheets and SnO_2_ nanoparticles as a comparison.

## Results and discussion

**Figure 1:**
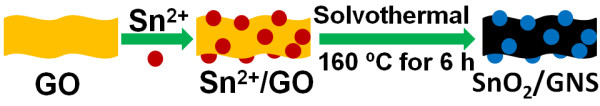
**Schematic diagram of the formation of SnO**_**2**_**/GNS nanocomposite.**

Figure 1 shows a schematic diagram of the formation of the SnO_2_/GNS nanocomposite. Firstly, Sn^2+^ ions were attracted to GO nanosheets in the ethylene glycol (EG) solution. Then, the anchored Sn^2+^ ions were reduced by EG via the following two-step reactions:

(1) $$2{\text{HOCH}}_{2}-{\text{CH}}_{2}\text{OH}\to 2{\text{CH}}_{3}\text{CHO}+2{\text{H}}_{2}\text{O}$$

(2) $$2{\text{CH}}_{3}\text{CHO}+{\text{SnCl}}_{}\to {\text{CH}}_{3}\text{CO}-{\text{COCH}}_{3}+\text{Sn}+2\text{HCl}$$

Simultaneously, GO nanosheets were also gradually reduced by EG to form graphene nanosheets. As EG is a mild reducing agent, the reduction processes take a long time (6 h) to complete at the high temperature (160°C). Then, the formed Sn nanoparticles were further oxidized by oxygen to become SnO_2_ nanoparticles during the cooling period. Consequently, the SnO_2_/GNS nanocomposite was obtained under the assistance of EG which acted not only as a dispersing agent but also as a reducing agent.

**Figure 2:**
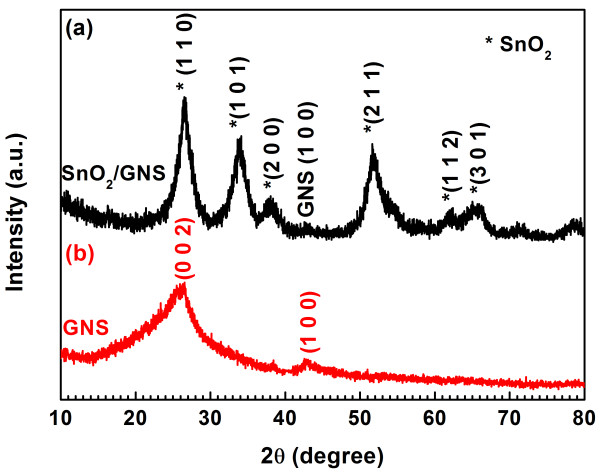
**XRD pattern of (a) SnO**_**2**_**/GNS nanocomposite and (b) GNS.**

XRD patterns of the SnO_2_/GNS nanocomposite and graphene were shown in Figure 2. In Figure 2a, X-ray diffraction lines of well-crystallized SnO_2_ were indexed to the tetragonal rutile SnO_2_ phase, which is consistent with the Joint Committee on Powder Diffraction Standards card 01–0657. The weak peak of graphene is also visible at 42° in the composite, which matches the diffraction peak (1 0 0) marked on the XRD pattern of bare graphene nanosheets (as shown in Figure 2b). XRD confirmed the coexisting phases of rutile SnO_2_ and graphene and the formation of SnO_2_/GNS nanocomposite.

**Figure 3:**
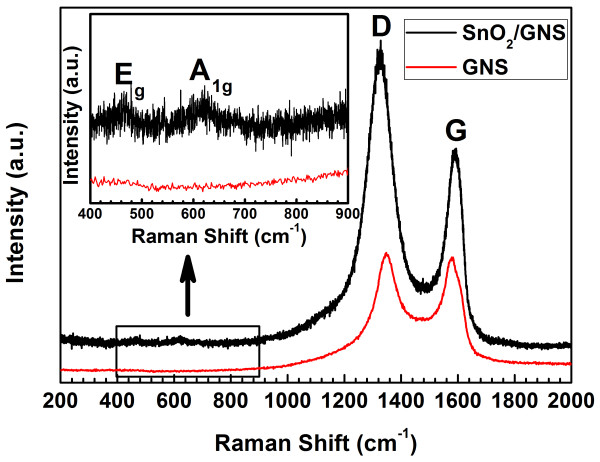
**Raman spectra of SnO**_**2**_**/GNS nanocomposite and bare GNS from 200 to 2,000 cm**^**−1**^**.** The inset shows magnified views of the spectra in the range of 400 to 900 cm^−1^.

**Figure 4:**
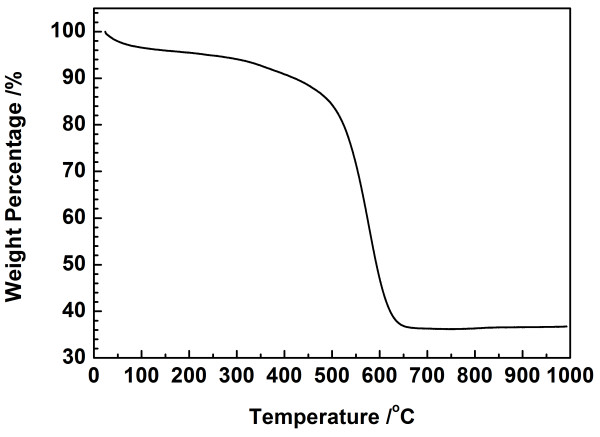
**TGA curve of SnO**_**2**_**/GNS nanocomposite.**

Raman spectra of the as-prepared SnO_2_/GNS nanocomposite and bare GNS are shown in Figure 3. It can be seen that both of them show high-intensity D band and G band at around 1,327 and 1,587 cm^−1^, respectively. The D band is stronger than the G band, and the D/G intensity ratio increased significantly compared to pristine graphite [23], which confirmed the existence of graphene nanosheets in the composite material. The D/G intensity ratio of the SnO_2_/GNS nanocomposite is higher than that of the bare GNS, indicating the decrease of the sp^2^ carbon domains when SnO_2_ nanoparticles were inserted between graphene nanosheets [24]. The inset in Figure 3 displayed magnified Raman spectra in the range of 400 to 900 cm^−1^ of both SnO_2_/GNS and bare GNS. Two weak peaks were found at 465 and 620 cm^−1^ in the Raman spectrum of the SnO_2_/GNS nanocomposite, which can be assigned to the E_g_ and A_1g_ active modes of SnO_2_ crystallines [25]. For the bare GNS, no Raman peak in this range was observed. TGA was employed to determine the weight composition of the SnO_2_/GNS nanocomposite (as shown in Figure 4). The dramatic weight loss from 500°C to 630°C is associated with the burning of graphene in air. SnO_2_ in the nanocomposite was stable up to 1,000°C. Therefore, the composition of the SnO_2_/GNS nanocomposite was calculated to be 36.3 wt% SnO_2_ and 63.7 wt% graphene.

**Figure 5:**
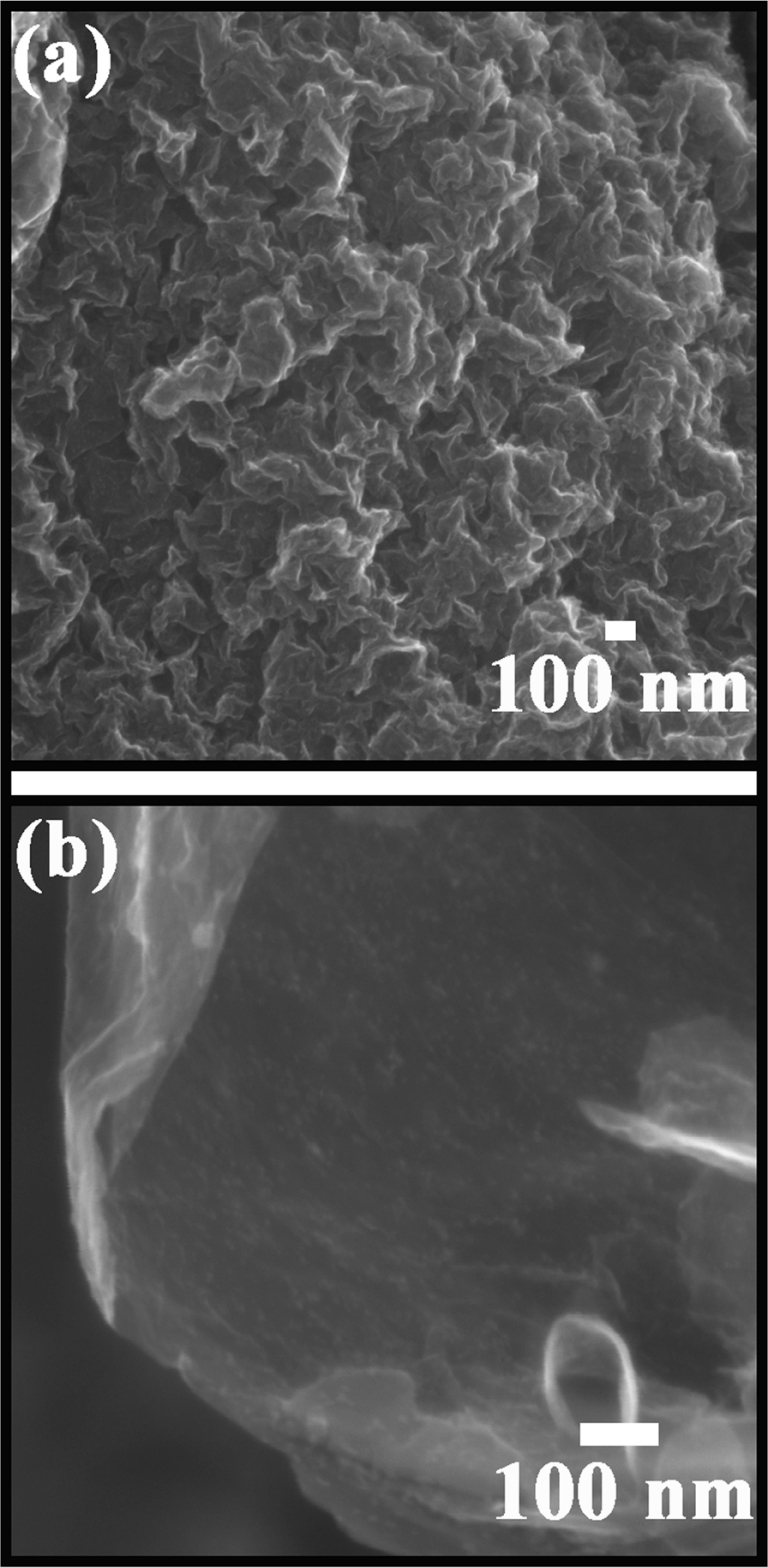
**SEM images of SnO**_**2**_**/GNS nanocomposite: (a) a low magnified image showing flower-like microstructure of graphene nanosheets and (b) a high magnified image focusing on a large graphene flake.**

**Figure 6:**
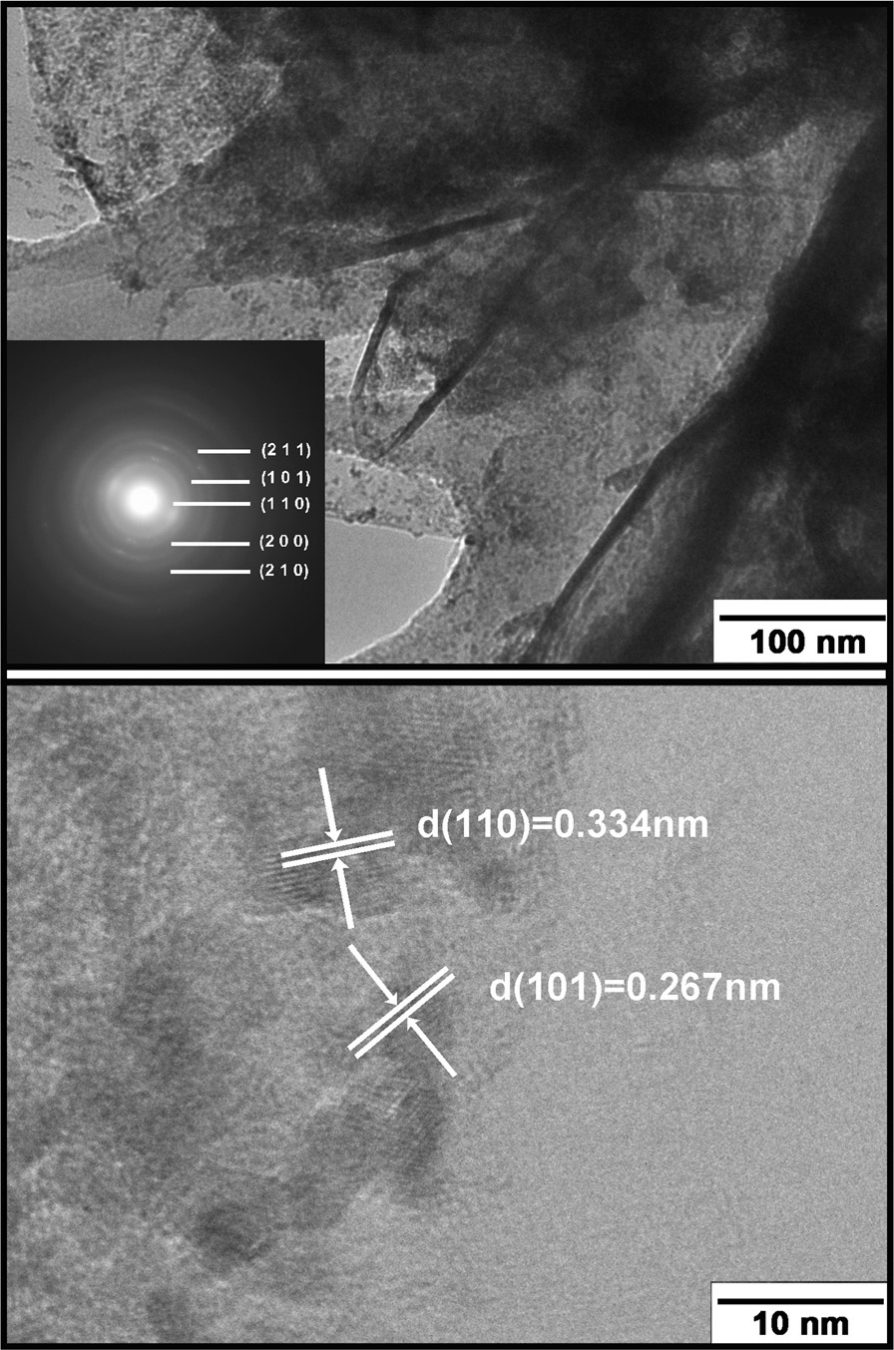
**TEM images of SnO**_**2**_**/GNS nanocomposite:*top*image shows a low magnified image exhibiting homogenous distribution of SnO**_**2**_**nanoparticles on graphene nanosheets.** The inset shows the corresponding SAED ring pattern, where crystal planes (1 1 0), (1 0 1), (2 0 0), (2 1 1), (2 1 0) of SnO_2_ are indexed; *bottom* image shows an HRTEM image focusing on a few SnO_2_ nanoparticles with indexed (1 0 0) and (1 1 0) crystal planes.

Figure 5 displays FESEM images of the SnO_2_/GNS nanocomposite. Corrugated graphene nanosheets are well expanded and form flower-like nanostructure (Figure 5a). A magnified scanning electron microscope (SEM) view further revealed details of a large, flat graphene nanosheet (Figure 5b). Tiny SnO_2_ nanoparticles were found anchored on this graphene flake. Figure 6 shows a TEM image of the SnO_2_/GNS nanocomposite. A large amount of SnO_2_ nanoparticles were homogeneously distributed on the graphene nanosheets as shown in Figure 6a. The inset shows the selected area electron diffraction pattern (SAED). The diffraction rings were indexed as the crystal planes (1 1 0), (1 0 1), (2 0 0), (2 1 1), (2 1 0) of SnO_2_, which clearly confirms the presence of SnO_2_ in the nanocomposite material. High resolution TEM (HRTEM) was performed on a few SnO_2_ nanoparticles (Figure 6b). SnO_2_ nanocrystals were densely packed on the surface of graphene nanosheets. Two crystal planes were indexed to be (1 0 0) and (1 1 0) of SnO_2_. The particle size of SnO_2_ was determined to be around 5 nm.

**Figure 7:**
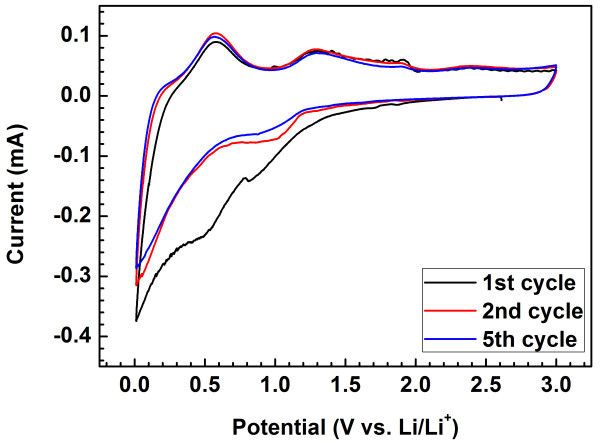
**CV curves of the SnO**_**2**_**/GNS nanocomposite electrode at the 1st, 2nd and 5th cycle at a sweep rate of 0.1 mV s**^**−1**^**.**

Figure 7 presents typical CV characteristics related to the lithiation and delithiation processes of the SnO_2_/GNS nanocomposite in the lithium-ion cell. A small cathodic peak appears at 0.8 V in the first cycle which can be attributed to the formation of the solid electrolyte interphase layer. Another small reduction peak located around 0.06 V could be due to the reactions between lithium and SnO_2_ nanoparticles to form Li_x_Sn alloys, while the insertion of lithium in graphene nanosheets could be identified as the reduction peak at 0.01 V. There are three oxidation peaks located around 0.13, 0.55, and 1.3 V, respectively. They correspond to different oxidation reactions during the charge process. The first anodic peak at 0.13 V represents the lithium extraction from graphene nanosheet. The 0.55 V oxidation peak can be assigned to the dealloying of Li_x_Sn, showing a reversible process. The third weak oxidation at 1.3 V could be resulted from the partial transformation of Sn metal to SnO_2_[26,27]. The high reversibility of the CV curves further confirmed the reversible redox reactions occurring in the lithium-ion cell between lithium and SnO_2_/GNS nanocomposite.

**Figure 8:**
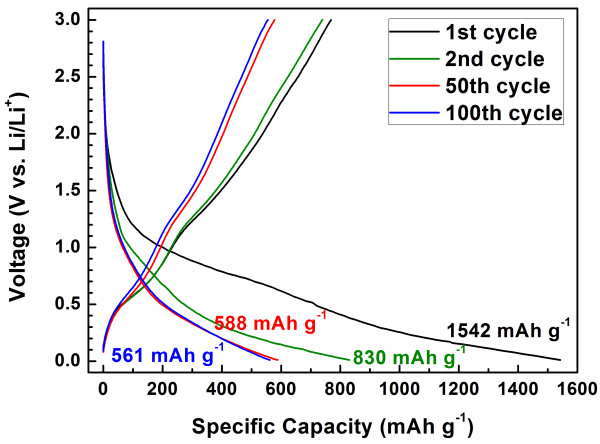
**Charge/discharge profiles of the SnO**_**2**_**/GNS nanocomposite electrode at a current rate of 0.1 C.**

**Figure 9:**
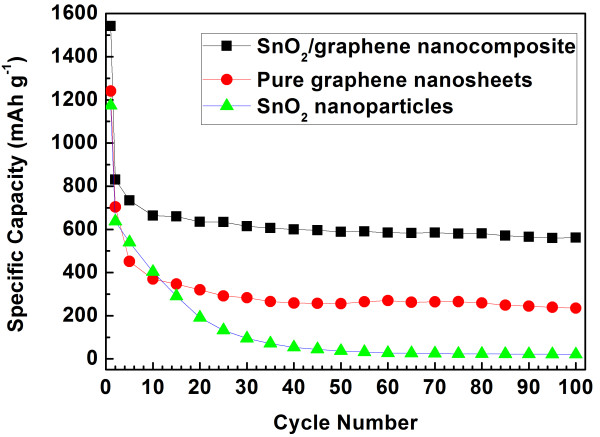
**Cycling performances of SnO**_**2**_**/GNS nanocomposite, GNS, and SnO**_**2**_**nanoparticles.**

**Figure 10:**
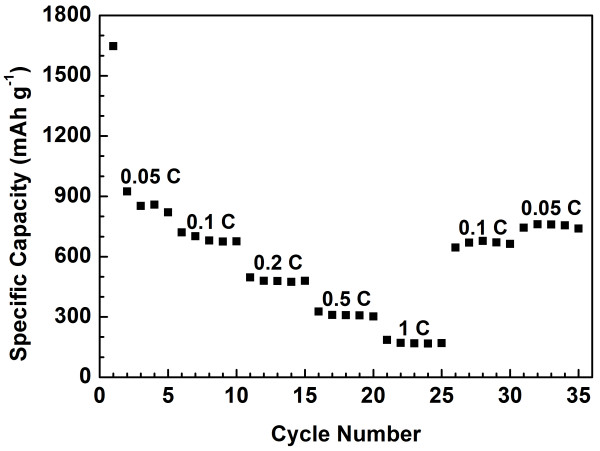
**Multiple-step cycling characteristic of the SnO**_**2**_**/GNS nanocomposite electrode at different current rates.**

Figure 8 shows the charge/discharge profiles of the SnO_2_/GNS nanocomposite electrode in different cycles at a current rate of 0.1 C. It can be seen that the SnO_2_/GNS electrode delivered a discharge capacity of 1,542 mAh g^−1^ in the first cycle. From the second cycle, the nanocomposite electrode exhibited highly reversible charge and discharge capacities. The maximum reversible discharge capacity of 830 mAh g^−1^ was achieved in the second discharge cycle. Specific discharge capacities of 588 and 561 mAh g^−1^ were obtained in the 50th and 100th cycle respectively, which indicates a very stable cycling performance. Significant improvement on the specific capacities has been achieved. Figure 9 shows the long-term cycling properties of the SnO_2_/GNS nanocomposite, bare graphene nanosheets and SnO_2_ nanoparticles at a 0.1 C current rate. The SnO_2_/GNS nanocomposite electrode demonstrated the highest reversible capacities and the best cycling stability. The nanocomposite electrode delivered a discharge capacity of 1,542 mAh g^−1^ in the first cycle and maintained stable capacities from the second cycle for 100 cycles with an excellent capacity retention. On the other hand, the bare GNS electrode showed a large irreversible capacity with lower reversible discharge capacities in 100 cycles. The capacities of SnO_2_ nanoparticles decrease quickly upon cycling. The retained capacity was less than 30 mAh g^−1^ in the 100th cycle. Figure 10 demonstrates multiple-step cycling characteristics of the SnO_2_/GNS nanocomposite electrode at 0.05 to 0.1, 0.2, 0.5, and 1 C and then reversing back to 0.1 and 0.05 C. The nanocomposite electrode was capable to deliver stable specific capacities at various current rates and recover substantial capacities without obvious capacity decline when returning to lower current rates. This indicated a fully reserved microstructure of the nanocomposite electrode after cycling at higher current rates.

**Figure 11:**
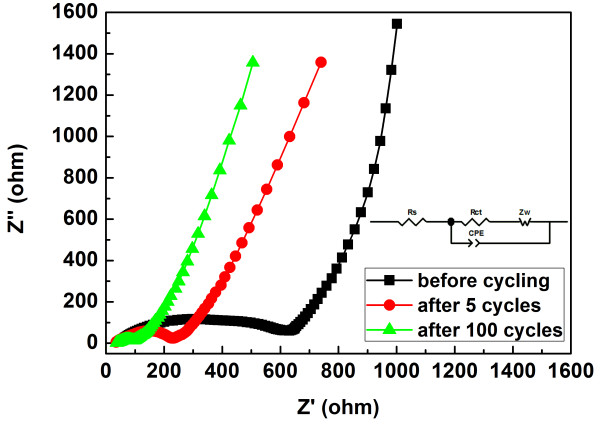
**AC impedance spectra of the SnO**_**2**_**/GNS nanocomposite electrode (a) before cycling, (b) after 5 cycles, and (c) after 100 cycles.** The inset shows the equivalent circuit model: *Rs* – resistance of the electrolyte; *Rct –* charge transfer resistance; *Zw –* Warburg resistance; *CPE* – constant phase element.

Figure 11 shows the alternating current (AC) impedance spectra of the SnO_2_/GNS nanocomposite electrode before cycling, after 5 cycles and after 100 cycles and the equivalent circuit model (inset). The intercept on the *Z*' axis at the high frequency region represents the resistance of the electrolyte (*R*_s_), which is 56.2 Ω for a fresh cell. The electrolyte resistance slightly decreased to 35 Ω after 5 cycles and remained nearly unchanged after 100 cycles (36.2 Ω). The diameters of the semicircles on the spectra implied the charger transfer resistances (*R*_ct_) at the electrolyte/electrode interface. It should be noted that the initial charge transfer resistance was 575.9 Ω then gradually decreased to 242.5 Ω (after 5 cycles) and 95.25 Ω (after 100 cycles) upon prolonged cycling. The significantly decreased charge transfer resistance could benefit for an enhanced cycle life of the SnO_2_/GNS nanocomposite electrode. The overall electrochemical performance of the SnO_2_/GNS nanocomposite was improved as graphene nanosheets supported SnO_2_ nanoparticles on their layered nanostructure. The inserted SnO_2_ nanoparticles reduce the stacking degree of graphene nanosheets and also contribute to the reversible lithium storage. Graphene nanosheets in the nanocomposite not only accommodate the volume change associated with the reactions between lithium and SnO_2_ nanoparticles, but also provide electrical conductance for the electrodes. For the SnO_2_/GNS nanocomposite prepared by the solvothermal method, the electrochemical properties were further improved due to the optimized nanoparticle distribution and small particle size of SnO_2_. The well-dispersed SnO_2_ nanoparticles effectively prevent the formation of agglomerates on graphene nanosheets, which induces an enhanced electrochemical performance.

## Conclusions

A facile solvothermal preparation method has been developed to synthesize the SnO_2_/GNS nanocomposite with a uniform nanoparticle distribution. The as-prepared SnO_2_/GNS nanocomposite exhibited an improved lithium storage capacity and cycling performance compared to bare GNS and bare SnO_2_ nanoparticles. The presence of GNS in the nanocomposite could increase the electrical conductivity and buffer the volume expansion associated with the lithiation and delithiation processes, leading to a significantly enhanced electrochemical performance. The solvothermal approach might be applicable for rapid and effective synthesis of other metal oxide/graphene nanocomposites.

## Abbreviations

GNS,, Graphene nanosheets; GO,, Graphene oxide.

## Competing interests

The authors declare that they have no competing interests.

## Authors' contributions

BW designed and carried out the experimental work, conducted basic characterizations of the sample, undertook all the electrochemical tests, analyzed all the data, and wrote the manuscript. DS performed the TEM observations. JP obtained the Raman spectra of the samples. AH and GW supervised the research work and GW critically revised the manuscript. All authors read and approved the final manuscript.
